# Lipoinjection with Adipose Stem Cells for Nasal Modeling: Rhino Cell, a Highly Versatile Alternative

**DOI:** 10.1055/a-2067-5481

**Published:** 2023-08-02

**Authors:** Yanko Castro-Govea, Jorge A. García-Garza, Sergio E. Vázquez-Lara, Cynthia M. González-Cantú, Hernán Chacón-Moreno, Víctor H. Cervantes-Kardasch

**Affiliations:** 1 Service of Plastic Surgery, School of Medicine and “Dr. Jose E. González” University Hospital, Autonomous University of Nuevo Leon, Monterrey, Nuevo Leon, Mexico; 2 Laboratory of Tissue Engineering, Faculty of Medicine, University of Colima, Colima, Mexico

**Keywords:** nonsurgical rhinoplasty, nasal modeling, ASCs, nasal lipoinjection, nanofat

## Abstract

It is undeniable that a significant number of patients who want to improve their facial appearance is increasingly interested in nonsurgical procedures. Without a doubt, the use of autologous fat could not be left out as a magnificent alternative for nasal modeling simply because of four influential factors: ease of collection, compatibility, the temporality of the results, and safety. This work describes an innovative alternative technique for nasal modeling using micrografts enriched with adipose-derived mesenchymal stem cells (ASCs). With this technique, fat was collected and divided into two samples, nanofat and microfat. Nanofat was used to isolate the ASCs; microfat was enriched with ASCs and used for nasal modeling. Lipoinjection was performed in a supraperiosteal plane on the nasal dorsum. Through a retrolabial access, the nasal tip and base of the columella were lipoinjected. We consider that nonsurgical nasal modeling using micrografts enriched with ASCs can be an attractive and innovative alternative. This technique will never be a substitute for surgical rhinoplasty. It can be performed in a minor procedure area with rapid recovery and return to the patient's daily activities the next day. If necessary, the procedure can be repeated.

## Introduction

Undeniably, a significant sector of patients who want to improve their facial appearance is increasingly interested in nonsurgical procedures. This trend currently represents a globalized perspective, not only for earlier recovery but also to quickly demonstrate the result without so much questioning or evidence that a surgical procedure can cause and logically with a lower cost.

The concept of nonsurgical rhinoplasty or rhinoplasty dates back to the mid-1980s. Different materials, including bovine collagen (heterogeneous), silicone, methyl methacrylate spheres, polytetrafluoroethylene, calcium hydroxyapatite, and hyaluronic acid (alloplastics), have been used. 1 New fillers and the knowledge obtained using these materials have made some obsolete over the years. Hyaluronic acid is probably the most widely used material as an alloplastic rhino-modeling agent. 2

Autologous facial fat injection is an excellent therapeutic resource in craniofacial and cosmetic surgery. 3 4 5 Autologous fat is a suitable alternative for nasal modeling because of four factors: ease of collection, compatibility, the temporality of the results, and safety. In this sense, various authors have reported good results using fat grafts for nasal modeling in cases such as radix complex, increased height of the nasal dorsum, saddle deformity, posttraumatic sequelae, etc. 6 7

For almost 20 years, in an effort to obtain higher survival rates for fat grafts, several authors have combined autologous fat grafting with adipose-derived mesenchymal stem cells (ASCs) for reconstructive and aesthetic purposes. 8 9 Traditionally, the stromal vascular fraction (SVF) is obtained through enzymatic digestion. SVF contains different cell types: endothelial cells, pericytes, preadipocytes, smooth muscle cells, macrophages, fibroblasts, and ASCs, all with the capacity for specialized differentiation. A huge window of opportunity in cell therapy was quickly visualized with this knowledge. 10

The invariable and continued trend for improving facial lipofilling has led to new techniques such as micrograft and nanograft and more competitive options to obtain a relatively greater degree of fat grafts permanence during contour remodeling facial rejuvenation. 9 These techniques have simplified enzymatic isolation by mechanically isolating the cell elements of the SVF to obtain a higher concentration representing a more practical and dynamic option for lipofilling. The differentiation capacity of the SVF cell conglomerate, including ASCs, makes the entire context of facial rejuvenation and tissue regeneration attractive and interesting.

Most autologous fat used in nonsurgical rhinoplasty is for nasal dorsum modeling. We consider a defined and projected nasal tip, including a borderline nasolabial angle with at least intermediate thickness skin, a priority for modeling the nasal tip.

Nasal modeling with autologous fat, enriched and not enriched with ASCs, will never be a substitute for conventional surgical rhinoplasty. However, we have found that a certain group of patients will not undergo surgical rhinoplasty for several reasons. We strongly believe that we must have innovative and competitive alternatives to satisfy the requests of our patients, who are increasingly younger.

This work describes an innovative idea for nasal modeling using micrografts enriched with ASCs. A 21-year-old female patient came to our clinic for a nasal dorsum and tip modeling. We describe this technique and a 12-month follow-up. Informed consent was obtained from the patient before the procedure.

## Idea

The patient is a 21-year-old woman with no relevant medical history. The procedure was performed under local anesthesia (dental cartridge: 2% lidocaine with epinephrine 1:100,000), similar to the supraperiosteal technique used by dentists to treat upper teeth. The needle was oriented retrolabially toward the columella base to achieve an anesthetic effect by blocking the terminal branches of the infraorbital nerve ascending toward the nasal tip and dorsum. Oral paracetamol and cefalexin were prescribed for 1 and 3 days after the procedure.

A tumescent and anesthetic solution of 10 mL lidocaine with 1% epinephrine in 20 mL saline solution was prepared. The solution was infiltrated in the central zone of the infraumbilical region. After 10 minutes, 10 mL of fat was collected using an 8-cm-long, 2.5-mm-diameter blunt-tipped multifenestrated cannula connected to a 10 mL Luer-Lok syringe. The microapproach was closed with a small round adhesive bandage.

### Fat Processing Technique

The collected fat was divided into sample 1 (5 mL) and sample 2 (5 mL). Both samples were processed according to the Coleman technique. 11 The supernatant and infranatant were subsequently removed. The first (nanofat) was used to isolate the ASCs; the second (microfat) was used for nasal modeling.

### Nanofat Processing for the Isolation of Adipose-Derived Mesenchymal Stem Cells

The nanofat harvest was conducted with cannulas from Tulip Medical (San Diego, CA) following Tonnard et al, 9 except we centrifuged the samples and increased the passages.

First, sample 1 was passed back and forth 40 times to another empty syringe using a 2.4-mm transfer connector, and second, the process was repeated with a 1.2-mm transfer connector. Third, the emulsified fat was passed once using a nanotransfer filter (500-µm mesh). The adipocytes are mechanically destroyed by emulsification of the fat with 80 passes. Filtration through the micromesh retained the residual fibrous tissue, isolating the cellular elements of the SVF.

### Adipose-Derived Mesenchymal Stem Cells –Enriched Microfat for Nasal Modeling

Nanofat content, 2.5 mL, is slowly transferred to sample 2 using a 4-mm transfer connector with three passes. This procedure was used to homogenize the microfat mixture with the cellular elements of the SVF.

### Rhino Modeling

**Fig. 1:**
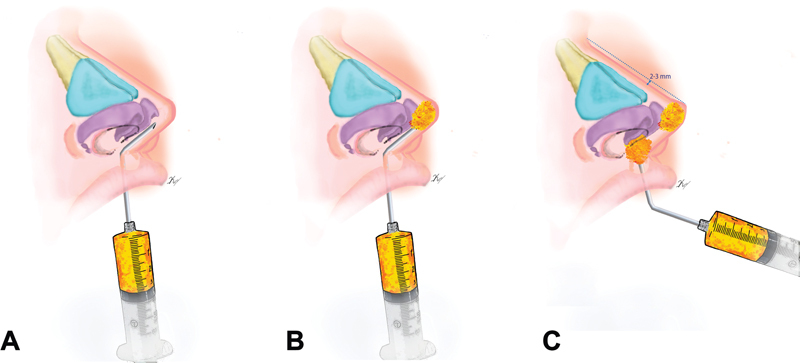
Rhino modeling. ( **A** ) Retrolabial introduction of the cannula angled toward the base of the columella and nasal tip. The cannula is held between the thumb and forefinger. ( **B** ) Lipoinjection of adipose-derived mesenchymal stem cells in the nasal tip. ( **C** ) The nasal tip is projected 2 to 3 mm above the height of the dorsum. Lipoinjection at the base of the columella helps maintain this increase in nasal tip projection.

A 3-mL syringe and a 10-cm-long, 1.7-mm-diameter malleable blunt-tipped cannula with a 2-mm beveled hole at the distal end were used. Intranasally, a marginal microapproach of 1 mm was performed lateral to the right dome. Initially, lipoinjection was performed on the nasal dorsum in a supraperiosteal plane from distal to proximal in an amount of 0.8 mL, which was digitally modeled to ensure uniform distribution. A 1-mm microapproach was subsequently made into the oral mucosa 0.5 mm anterior to the upper frenulum with the upper lip everted. The malleable cannula, previously angled at approximately 45° degrees, was directed in the last four centimeters toward the columella base, holding it with the thumb and forefinger, where we feel the cannula between our fingers. It continues to be inserted safely up to the nasal tip ( Fig. 1A ).

We prefer to initially lipoinject 0.3 mL to control the final amount required (0.6 mL total; Fig. 1B ). Specifically, the nasal tip was projected approximately 2 to 3 mm above the height of the nasal dorsum; another 0.3 mL was injected to obtain this effect.

**Fig. 2:**
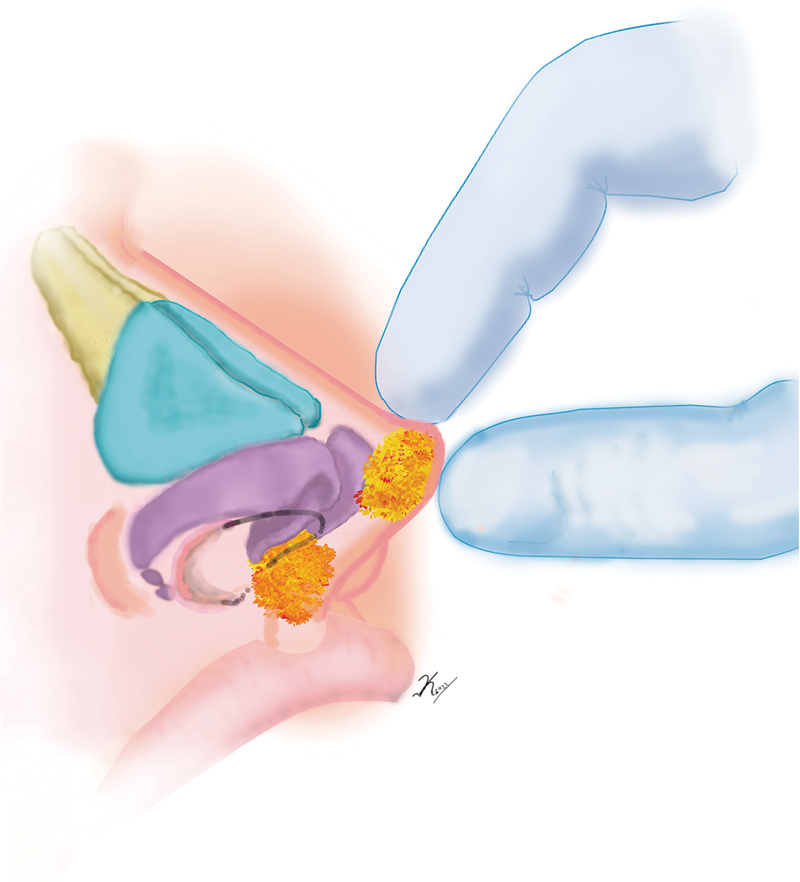
Finally, we digitally model the injected fat to ensure a somewhat hyperprojected nasal tip.

**Fig. 3:**
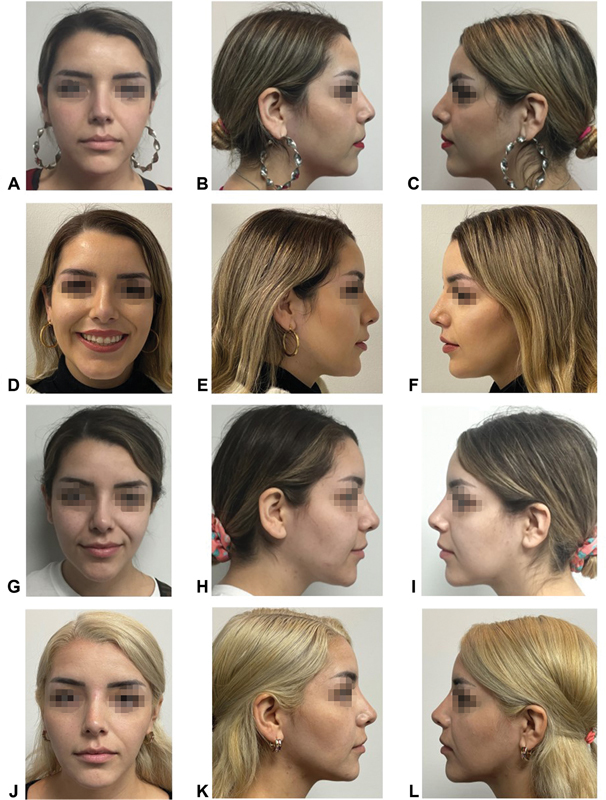
The sequence of the follow-up period before and after rhino modeling. ( **A, B, C** ) Preoperative view. ( **D, E, F** ) Postoperative view with 3 months of evolution. ( **G, H, I** ) Postoperative view with 6 months of evolution. ( **J, K, L** ) Postoperative view with 12 months of evolution.

In this sense, 0.6 mL was also lipoinjected at the columella base to help maintain the increase in the projection of the nasal tip ( Fig. 1C ). We digitally model the injected fat to ensure a somewhat hyperprojected nasal tip ( Fig. 2 ). Finally, we place nasal tape to contain the modeling to a certain degree with the understanding that we have injected a soft material. This tape is removed in 3 days. The results of this technique remained stable. The results were documented photographically in a follow-up period of 3 to 12 months with no complications ( Fig. 3 ).

## Discussion

Autologous fat graft (AFG) is a good option to camouflage irregularities in the nasal dorsum during and after surgical rhinoplasty. 12 In the same way, it is also a great resource to provide skin thickening, particularly in secondary or tertiary rhinoplasties, characterized by great tissue thinness, revealing irregularities of the cartilaginous bone framework of the nasal dorsum. 13

It is important to note that we must have pertinent anatomical knowledge and follow a safety protocol to minimize the possibility of vascular and ocular compromise. A fundamental safety step is using a blunt-tipped cannula with a diameter larger than blood vessels. We then perform lipoinjection slowly and retrogradely in a supraperiosteal plane, which is mostly avascular. Lipoinjection of the nasal tip is conducted with a cannula angled in a sub-Superficial Musculoaponeurotic System (sub-SMAS) plane.

An interesting study by Kao et al 14 reported that autologous fat microtransplantation is an adequate strategy to augment the nasal dorsum with an acceptable degree of patient satisfaction after 1 to 3 sessions and with an average follow-up period of almost 2 years. A systematic review of AFG for augmentation rhinoplasty concluded that autologous fat rhinoplasty is a safe treatment, with high satisfaction rates and minimal complications, 63 and 100%, respectively. 15

We have not found specific information on rhinoplasty with autologous fat enriched with ASCs. The tissue regeneration capacity of the cellular elements of SVF, within them the ASCs, is largely due to vascular endothelial growth factor, growth factor, and transforming growth factor b, which contribute to stem cell differentiation, in turn favoring the proliferation of endothelial cells, blood vessels, new adipocytes, and mesenchymal stem cells, among others. 16 17 We know what the unpredictability of the permanence and stability of fat grafts represents; however, we believe that the use of ASCs at higher concentrations seems to favor better conditions for the differentiation of the different types of cellular elements and, therefore, obtaining relatively better graft retention rates and, logically, greater permanence. 18 19 20

In rhinoplasty with alloplastic fillers, the patient normally knows that this procedure lasts a certain time, so it must be repeated to maintain the result. It is important to explain to the patient the possibility of performing a second lipoinjection session to obtain better long-term stability.

We usually establish a 6- to 9-month follow-up period where we can assess the degree of permanence and therefore the rate of absorption; we consider that when you perform this procedure with the ideal conditions the result will mostly be good, otherwise, the result will not be as expected. We know the advantages of modeling the nasal dorsum and, to a certain degree, the nasal length. However, we prioritize specific patient conditions when modeling the nasal tip. A nasal tip with some mestizo characteristics and thick covering tissue can be modeled, but the nasal definition and projection improvement will be poor. Therefore, as we mentioned before, we think that the most favorable conditions to obtain better modeling of the nasal tip are reasonably developed and resistant lower lateral cartilages, which favor a borderline degree of definition and projection of the nasal tip, and, logically, a skin thickness that is at least moderately thin.

We consider that nonsurgical nasal modeling using micrografts enriched with ASCs can be an innovative alternative. Its versatility provides advantages for aesthetic nasal modeling and as a resource to correct other types of nasal asymmetry.

This technique will never be a substitute for surgical rhinoplasty. We believe that we must have other options in the growing demand from patients who request nonsurgical nasal modeling. This procedure can be performed in a minor surgery area with rapid recovery and return to the patient's daily activities the next day. Success will depend on whether we have the right patient, and of course, on the surgeon's experience and creativity. It is a safe and facially reproducible option that can be performed in less than an hour. The procedure can be repeated in case of need. Nevertheless, more studies are needed to reinforce the stability of the long-term results.

## References

[1] Sánchez-Carpintero I Candelas D Ruiz-Rodríguez R [Dermal fillers: types, indications, and complications] Actas Dermosifiliogr 2010 101 05 381 393 20525480 10.1016/s1578-2190(10)70660-0

[2] Attenello N H Maas C S Injectable fillers: review of material and properties Facial Plast Surg 2015 31 01 29 34 25763894 10.1055/s-0035-1544924

[3] Guerrerosantos J Guerrerosantos F Orozco J Classification and treatment of facial tissue atrophy in Parry-Romberg disease Aesthetic Plast Surg 2007 31 05 424 434 17700981 10.1007/s00266-006-0215-4

[4] Castro-Govea Y Vela-Martinez A Treviño-Garcia L A Lipoinjection and multiple internal cuts for congenital constriction bands: a new treatment approach Aesthetic Plast Surg 2017 41 02 375 380 28035448 10.1007/s00266-016-0744-4 PMC5371641

[5] Gornitsky J Viezel-Mathieu A Alnaif N Azzi A J Gilardino M S A systematic review of the effectiveness and complications of fat grafting in the facial region JPRAS Open 2018 19 87 97 32158860 10.1016/j.jpra.2018.12.004 PMC7061561

[6] Kornstein A N Nikfarjam J S Fat grafting to the forehead/glabella/radix complex and pyriform aperture: aesthetic and anti-aging implications Plast Reconstr Surg Glob Open 2015 3 08 e500 26495213 10.1097/GOX.0000000000000470 PMC4560233

[7] Lin S Hsiao Y C Huang J J Minimal invasive rhinoplasty: fat injection for nasal dorsum contouring Ann Plast Surg 2017 78(3, suppl 2):S117 S123 28195887 10.1097/SAP.0000000000001016

[8] Castro-Govea Y De La Garza-Pineda O Lara-Arias J Cell-assisted lipotransfer for the treatment of Parry-Romberg syndrome Arch Plast Surg 2012 39 06 659 662 23233894 10.5999/aps.2012.39.6.659 PMC3518012

[9] Tonnard P Verpaele A Peeters G Hamdi M Cornelissen M Declercq H Nanofat grafting: basic research and clinical applications Plast Reconstr Surg 2013 132 04 1017 1026 23783059 10.1097/PRS.0b013e31829fe1b0

[10] Zuk P A Zhu M Mizuno H Multilineage cells from human adipose tissue: implications for cell-based therapies Tissue Eng 2001 7 02 211 228 11304456 10.1089/107632701300062859

[11] Coleman S R Structural fat grafts: the ideal filler? Clin Plast Surg 2001 28 01 111 119 11248861

[12] Cárdenas J C Carvajal J Refinement of rhinoplasty with lipoinjection Aesthetic Plast Surg 2007 31 05 501 505 17653684 10.1007/s00266-006-0136-2

[13] Baptista C Nguyen P S Desouches C Magalon G Bardot J Casanova D Correction of sequelae of rhinoplasty by lipofilling J Plast Reconstr Aesthet Surg 2013 66 06 805 811 23566743 10.1016/j.bjps.2013.02.020

[14] Kao W P Lin Y N Lin T Y Microautologous fat transplantation for primary augmentation rhinoplasty: long-term monitoring of 198 Asian patients Aesthet Surg J 2016 36 06 648 656 26764261 10.1093/asj/sjv253 PMC5127412

[15] Keyhan S O Ramezanzade S Bohluli B Fallahi H R Mirzahoseini S Nahai F Autologous fat injection for augmentation rhinoplasty: a systematic review Aesthet Surg J Open Forum 2021 3 02 ojab010 33987531 10.1093/asjof/ojab010 PMC8092143

[16] Kølle S F Fischer-Nielsen A Mathiasen A B Enrichment of autologous fat grafts with ex-vivo expanded adipose tissue-derived stem cells for graft survival: a randomised placebo-controlled trial Lancet 2013 382(9898):1113 1120 24075051 10.1016/S0140-6736(13)61410-5

[17] Kuno S Yoshimura K Condensation of tissue and stem cells for fat grafting Clin Plast Surg 2015 42 02 191 197 25827563 10.1016/j.cps.2014.12.006

[18] Rihani J Microfat and nanofat: when and where these treatments work Facial Plast Surg Clin North Am 2019 27 03 321 330 31280846 10.1016/j.fsc.2019.03.004

[19] Cohen S R Tiryaki T Womack H A Canikyan S Schlaudraff K U Scheflan M Cellular optimization of nanofat: comparison of two nanofat processing devices in terms of cell count and viability Aesthet Surg J Open Forum 2019 1 04 ojz028 33791619 10.1093/asjof/ojz028 PMC7780476

[20] Tonnard P Verpaele A Carvas M Fat grafting for facial rejuvenation with nanofat grafts Clin Plast Surg 2020 47 01 53 62 31739897 10.1016/j.cps.2019.08.006

